# Structural Modulation of Brain Development by Oxygen: Evidence on Adolescents Migrating from High Altitude to Sea Level Environment

**DOI:** 10.1371/journal.pone.0067803

**Published:** 2013-07-09

**Authors:** Jiaxing Zhang, Haiyan Zhang, Ji Chen, Ming Fan, Qiyong Gong

**Affiliations:** 1 Department of Physiology and Neurobiology, Medical College of Xiamen University, Xiamen, China; 2 Department of Physiology, Weifang Nursing Vocational College, Weifang, China; 3 Department of Brain Protection and Plasticity, Institute of Basic Medical Sciences, Beijing, China; 4 Department of Radiology, Huaxi Magnetic Resonance Research Center (HMRRC), West China Hospital, Sichuan University, Chengdu, China; Beijing Normal University, China

## Abstract

The present study aimed to investigate structural modulation of brain by high level of oxygen during its peak period of development. Voxel-based morphometry analysis of gray matter (GM) and white matter (WM) volumes and Tract-Based Spatial Statistics analysis of WM fractional anisotropy (FA) and mean diffusion (MD) based on MRI images were carried out on 21 Tibetan adolencents (15–18 years), who were born and raised in Qinghai-Tibetan Plateau (2900–4700 m) and have lived at sea level (SL) in the last 4 years. The control group consisted of matched Tibetan adolescents born and raised at high altitude all the time. SL immigrants had increased GM volume in the left insula, left inferior parietal gyrus, and right superior parietal gyrus and decreased GM in the left precentral cortex and multiple sites in cerebellar cortex (left lobule 8, bilateral lobule 6 and crus 1/2). Decreased WM volume was found in the right superior frontal gyrus in SL immigrants. SL immigrants had higher FA and lower MD at multiple sites of WM tracts. Moreover, we detected changes in ventilation and circulation. GM volume in cerebellum lobule 8 positively correlated with diastolic pressure, while GM volume in insula positively correlated vital capacity and hypoxic ventilatory response. Our finding indicate that the structural modulations of GM by high level of oxygen during its peak period of development are related to respiratory and circulatory regulations, while the modulation in WM mainly exhibits an enhancement in myelin maturation.

## Introduction

The human brain structures have distinct developmental trajectories [1]. Total cerebral volume follows an inverted U shaped trajectory peaking at age 10.5 in girls and 14.5 in boys [2]. Total cerebellum volume also follows an inverted U shaped developmental trajectory peaking at 11.3 in girls and 15.6 in boys [3]. Gray matter (GM) volumes in various brain regions follow an inverted U developmental trajectory with a peak at age 12–17, while white matter (WM) volumes steadily increase throughout childhood and the increase continues during the twenties in several association tracts [1]. Fractional anisotropy (FA) and mean diffusion (MD) show more rapid changes during adolescence (increases for FA, decreases for MD) and slower changes or levelling off during young adulthood [4]. Throughout the lifespan, the human brain is continuously shaped by environmental factors [5]. If environmental stresses occur during these critical developmental periods they might have a great impact on brain maturation.

Every year there are increasing amount of high altitude (HA) native adolescents immigrating to lowlands due to study, and stay for several years. These HA born and grown residents have distinctive biological characteristics in cerebral metabolism [6], cerebral autoregulation [7], cerebral blood flow velocity [8], cerebrovascular reactivity [9], and brain function and morphology [10] to offset chronic hypoxia. The brain is one of the heaviest oxygen consumers in the body [11]. When these HA residents move to lowlands during their developmental stage, oxygen-enriched air inhalation would increase their brain oxygen supply and tissue oxygen concentration [12], and thus the brain inevitably suffers from oxidative stress.

Hyperoxic exposure during development leads to neuronal cell death [13]–[18]. However, hyperoxia can also induce neurogenesis if it is controlled at low-to-moderate levels [19]. Hyperoxia was found to change CMRO2 [20], [21], which may contribute to neurogenesis or neurodegeneration. Hyperoxia also cause a redistribution of cerebral blood flow (CBF) [22]–[24], which would change oxygen supply. In some brain regions, neuronal activities can be enhanced by transient inhalation of high concentration of oxygen [25]–[28]. In HA residents who have immigrated to lowlands for a long period of time, peripheral physiological systems typically employ adaptive mechanisms such as alterations in respiratory and circulatory function [29]–[32] and hemoglobin concentration [33]. Such alterations change oxygen transport in the cerebral blood flow, and then lead to cumulative changes in brain structure. Moreover, the brain is the control centre of the body. At lowlands, the adaptation in the cardiovascular and respiratory systems may act on their control centers in the brain through afferent feedback. Taken together, these literatures suggest the brain structure would be largely affected by high concentration of oxygen exposure. However, until now, it remains largely uninvestigated.

In the present study, HA adolescents who have immigrated to sea level (SL) for 4 years were recruited for this purpose. Quantitative analysis methods such as voxel-based morphometry (VBM) and Tract-Based Spatial Statistics (TBSS) based on MRI data were employed to measure GM and WM microstructural changes. Recently, the preprocessing steps of VBM have been improved with the Diffeomorphic Anatomical Registration Through Exponentiated Lie algebra (DARTEL) registration method [34], which can achieve more accurate inter-subject registration of brain images. TBSS uses diffusion tensor MR imaging (DTI) to measure differences in FA and MD between groups [35]. FA represents the ratio between the length of the primary axis and the other two orthogonal axes. High anisotropy would represent diffusion that is highly oriented in one direction. MD represents the overall free space available for the water to self-diffuse, and thus is the average length of all the three axes [36]. These two methods have been used in our recent studies on HA immigrant descendants and mountain climbers [10], [37].

A few of fMRI studies have revealed that a number of brain regions, such as insula, cerebellum, sensory and premotor areas, respond to inhalation of high concentration of oxygen [25]–[28]. The WM microstructural alterations have been shown in a number of regions; most prominent in the corpus callosum, corticospinal tract, and cerebellum across HA exposed population [10], [37]. Therefore, we hypothesized that GM and WM in these brain regions would be modulated by high level of oxygen. HA natives have developed adaptations in respiratory and cardiovascular function [38]–[40]. After residing at SL for a long period of time, they redeveloped an adaption to high concentration of oxygen environment [29]–[32], [41], [42]. In the present study, pulmonary function and cardiovascular function were also examined. We further hypothesized that the structural modifications in these brain regions may underlie the respiratory and cardiovascular alterations in HA adolescents living at SL. Respiratory adaptation to HA mainly characterized by blunting to hypoxic ventilatory response (HVR) [43]. However, HA natives who are resident at SL had a relatively normal acute HVR [44]. The insular cortex has been suggested to play an important role in the unpleasantness of dyspnea [45]. In the present study, HVR was also examined and we expected that the GM change in insula may clarify the mechanism involved in SL HVR.

## Materials and Methods

### Subjects

The subjects were 21 Tibetan adolescents who were born and raised at the altitude of 2900–4700 m in Qinghai-Tibetan Plateau and have attended middle school in SL in the last 4 years. During their time at SL they have never returned to HA. The control subjects were 21 Tibetan adolescents born and raised at HA all the time. Currently, the two groups have been enrolled in the same high school at Chengdu (<400 m) for half a month. SL immigrants and controls were classmates and did not differ in high school enrollment scores. In addition, the control subjects were matched with SL immigrants by gender, age, the altitude that they have been living at HA, and the family socioeconomic status. All subjects were right-handed, and were excluded if they had (1) chronic mountain sickness, (2) a documented neurological disorder, (3) a past history of head injury with loss of consciousness, or (4) serious “high altitude deadaptation reaction” at SL [46]. Procedures were fully explained, and all subjects provided written informed consent before participating in the study. The experimental protocol was approved by the Research Ethics Review Board of Xiamen University.

### Physiological Tests

Physiological tests were conducted 1 day before the MRI scan. The tests included arterial blood pressure measures, arterial blood gas analysis, and pulmonary function measure. HVR was assessed by using an acute protocol. The subjects breathed through a face mask, which was connected to a bottle (2000 ml) containing soda lime (absorbing CO2). This protocol progressively induce hypoxia over 1–2 min. HVR was calculated as the change of tide volume (ΔT)/the change of SaO2 (ΔSaO2) ratio after breath of hypoxia. All data were analyzed using SPSS 19.0. Independent t test was adopted to measure between-group differences. Statistical significance was set at p<0.05 (corrected for age).

### MRI Data Acquisition

MRI scans were conducted on the first day till 15th day after all subjects’ arrival at Chengdu. Structural images were acquired on a GE 3.0 T GE Signa EXCITE scanner (General Electric, Milwaukee, WI, USA) at Huaxi Magnetic Resonance Research Center (HMRRC, West China Hospital, Chengdu, China). A 3D structural MRI was acquired using a T1-weighted MPRAGE sequence (TR/TE = 8.5 ms/3.4 ms, FOV = 28×28 cm^2^, NEX = 1, in-plane resolution = 0.5476×1.094 mm^2^, flip angle = 12°, slice thickness = 1 mm). Conventional 2D T1 and T2 images were also acquired. A DTI pulse sequence with single shot diffusion-weighted echo planar imaging (TR/TE = 10000/70.8 ms, FOV = 24×24 cm^2^, in-plane resolution = 1.875×1.875 mm^2^, slice thickness = 3 mm) was applied sequentially in 16 different directions. The data analysis was conducted by two researchers who were blind to the status of subjects.

### VBM Analysis of GM and WM Volume

Data were analyzed using VBM8 toolbox implemented in SPM8 (Welcome Department of Imaging Neuroscience, University College London, London, UK). Calculations and image matrix manipulations were performed by using MATLAB (MathWorks, Natick, Massachusetts). The processes included the following steps: (i) the images were inspected and set at the anterior commissure. Each reorientated image was segmented into GM, WM, and cerebrospinal fluid (CSF) in native space and Procrustes aligned GM and WM images were generated by a rigid transformation. (ii) the DARTEL was used to create study-specific template by the aligned images from all the subjects to improve inter-subject registration of structural images [34]. The procedure implicated in six iterations, which began with the averaging of aligned data to generate an original template. Then, the first iteration of the registration was done on each subject and a new template was created. After this, the second iteration began. When six iterations were finished, the template was generated, which was the average of the DARTEL registered data. During iterations, all images were warped to the template yielding a series of flow fields that parameterized deformations in order to use in modulation to preserve actual GM and WM volume. The GM and WM images were modulated to account for the local compression and stretching that occurs as a consequence of the warping and affine transformation which is based on the change of variables theorem. (iii) the normalized images were transformed into MNI space. These GM and WM images were then smoothed using a Gaussian kernel of 8 mm full-width at half-maximum. Independent t-tests were performed to examine between-group differences, using gender, age, and total intracranial volume as covariates. The statistical parametric map was generated at t>3.3256, p<0.001 (uncorrected for multiple comparisons).

GM values in the changed regions were extracted from individual’s normalized and smoothed GM maps. Partial correlations (controlling for gender, age, and total intracranial volume) were then calculated between GM volumes of the clusters with group differences and physiological data. Statistical significance was set at p<0.05.

### TBSS Analysis of FA and MD

DCM2NII was used to convert diffusion tensor images from the proprietary scanner format to the NIFTI format. Then the images were processed using the FSL 4.1.5 software package (http://www.fmrib.ox.ac.uk/fsl/). Detailed processes were described in our previous studies [37]. TBSS processing includes the following steps: (i) align the FA images of all subjects to a template which was arbitrarily selected from those FA images by nonlinear registrations; (ii) transform all the aligned FA images into 1×1×1 mm^3^ MNI152 space by affine registrations to remove the effect of cross-subject spatial variability that remains after the non-linear registration; (iii) create the mean FA image and filter to retain only the centre of the WM tracts, with the threshold FA ≥0.20, and successfully exclude voxels, which consisted of GM or cerebrospinal fluid in the majority of subjects, so as to create the mean FA skeleton. (iv) project individual subjects’ FAs onto mean FA skeleton. (v) following these steps, data was fed into voxel-wise cross-subject statistical analyses. In all cases, the null distribution was built up over 5000 permutations. Independent t-tests were performed to examine between-group differences, using gender and age as covariates. The statistical parametric map was generated at p<0.05 and p<0.005, respectively (uncorrected for multiple comparisons).

The same TBSS processing was used for MD analysis; the statistical parametric map was generated at p<0.001, false discovery rate (FDR) corrected for multiple comparisons.

## Results

### Physiological Data

The physiological values are shown in Table 1. SL immigrants had a significantly lower value in systolic pressure and diastolic pressure and a higher value in oxygen saturation and ΔT/ΔSaO2 than controls. Moreover, in males, SL immigrants had a higher vital capacity than controls.

**Table 1:** Demographic and physiological characteristics.

	SL immigrants	HA residents	P
Number of subjects	21	21	
Gender (male/female)	6/15	6/15	
Age (yr)	16.5±0.7 (15–18)	16.3±0. 8 (15–18)	0.203
HA location (m)	before/recent 4 yrs:		
	3750.6±495.9/<300	3756.4±433.1	0.966
SaO2 (%)	98.3±0.9	97.1±1.3	0.002
Blood pressure (mmHg)			
systolic pressure	113.7±9.0	121.0±13.4	0.046
diastolic pressure	65.5±7.9	72.6±12.0	0.032
Vital capacity (liters)			
(male/female)	5.0±1.0/3.3±0.3	3.8±0.7/3.2±0.5	0.048/0.488
Respiratory rate (times/min)			
(male/female)	19.5±4.2/19.2±1.3	20.3±1.5/20.1±1.7	0.659/0.107
HVR (ΔT/ΔSaO2)	60.2±26.8	39.1±22.4	0.008

HVR, hypoxic ventilatory response; SaO2, oxygen saturation. Data are shown as mean ± SD.

### Total Volume of GM, WM, and CSF

No subject from either group showed visible abnormalities on T1-weighted structural images. There were no significant group differences in total GM (SL immigrants vs. controls: 660.8±46.6 cm^3^ vs. 683.3±85.2 cm^3^, p = 0.296), WM (494.8±42.6 cm^3^ vs. 494.2±51.5 cm^3^, p = 0.969), and CSF (184.5±18.3 cm^3^ vs. 192.8±22.8 cm^3^, p = 0.205) volume.

### Regional GM Volume

**Figure 1:**
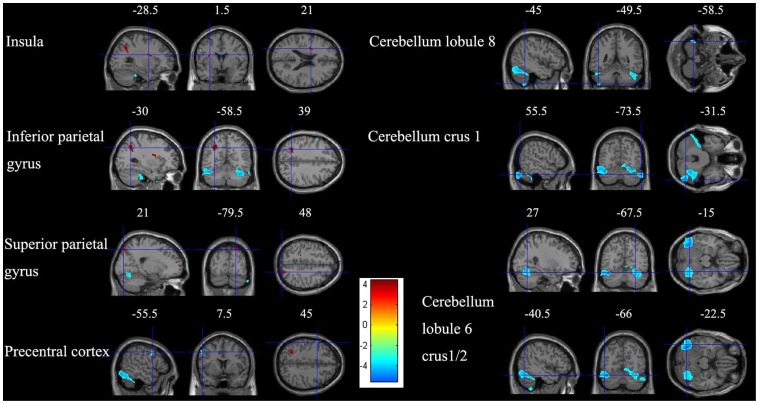
Gray matter volume changes in sea level immigrants versus high altitude residents. Sections (sagittal, coronal, and axial view) depicting regions showing increased regional gray matter volume in the left insula, left inferior parietal gyrus, and right superior parietal gyrus (red) and decreased gray matter in the left precentral cortex, left cerebellum lobule 8, and bilateral cerebellum lobule 6, crus 1 and crus 2 (blue) (p<0.001, uncorrected).

Compared with controls, SL immigrants had increased regional GM volume (clusters size >30 mm^3^) in the left insula, left inferior parietal gyrus, and right superior parietal gyrus; SL immigrants had decreased regional GM volume in the left precentral cortex (mainly including middle frontal gyrus), left cerebellum lobule 8, and bilateral cerebellum lobule 6, crus 1 and crus 2 (Figure 1, Table 2).

**Table 2:** Regional information of changed gray and white matter volume.

Areas	Volume (mm^3^)	Brodmann areas	MNI coordinate			t-score (peak)
			x	y	z	
***Gray matter***						
Insula_L	77	13	−28.5	1.5	21	3.7667
Parietal_Inf_L	295	7	−30	−58.5	39	4.3940
Parietal_Sup_R	127	7	21	−79.5	48	3.6769
Cerebelum_8_L	142		−45	−49.5	−58.5	3.5535
Cerebelum_6/Crus1/Crus2_R	3182		27	−67.5	−15	4.7251
Cerebelum_6/Crus1_L	3237		−40.5	−66	−22.5	5.4801
Cerebelum_Crus1_R	790		55.5	−73.5	−31.5	4.6777
Precentral_L	81	8	−55.5	7.5	45	3.5529
						
***White matter***						
Frontal_Sup_R	80	9	7.5	58.5	37.5	3.4400

### Regional WM Volume

**Figure 2:**
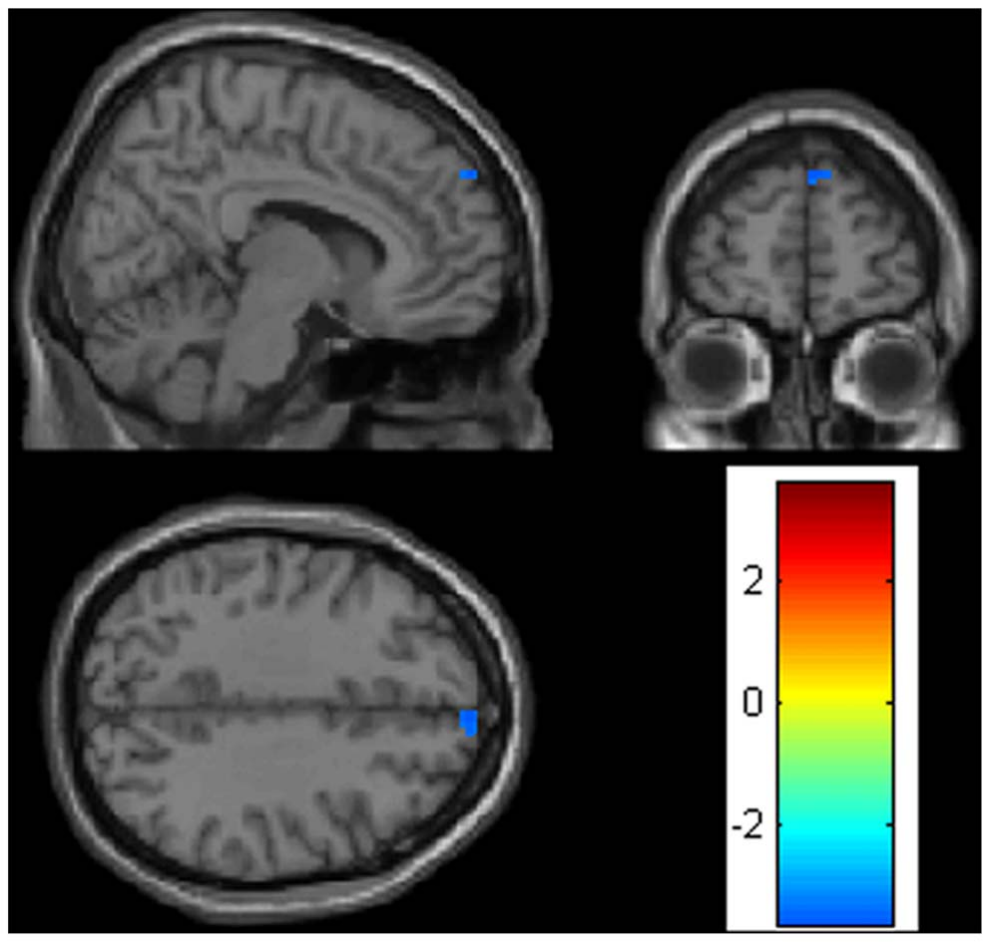
White matter volume changes in sea level immigrants versus high altitude residents. Sections (sagittal, coronal, and axial view) depicting regions showing decreased white matter in the right superior frontal gyrus (p<0.001, uncorrected).

Compared with controls, SL immigrants had decreased regional WM volume (clusters size >30 mm^3^) in the right superior frontal gyrus (Figure 2, Table 2).

### FA Values

**Figure 3:**
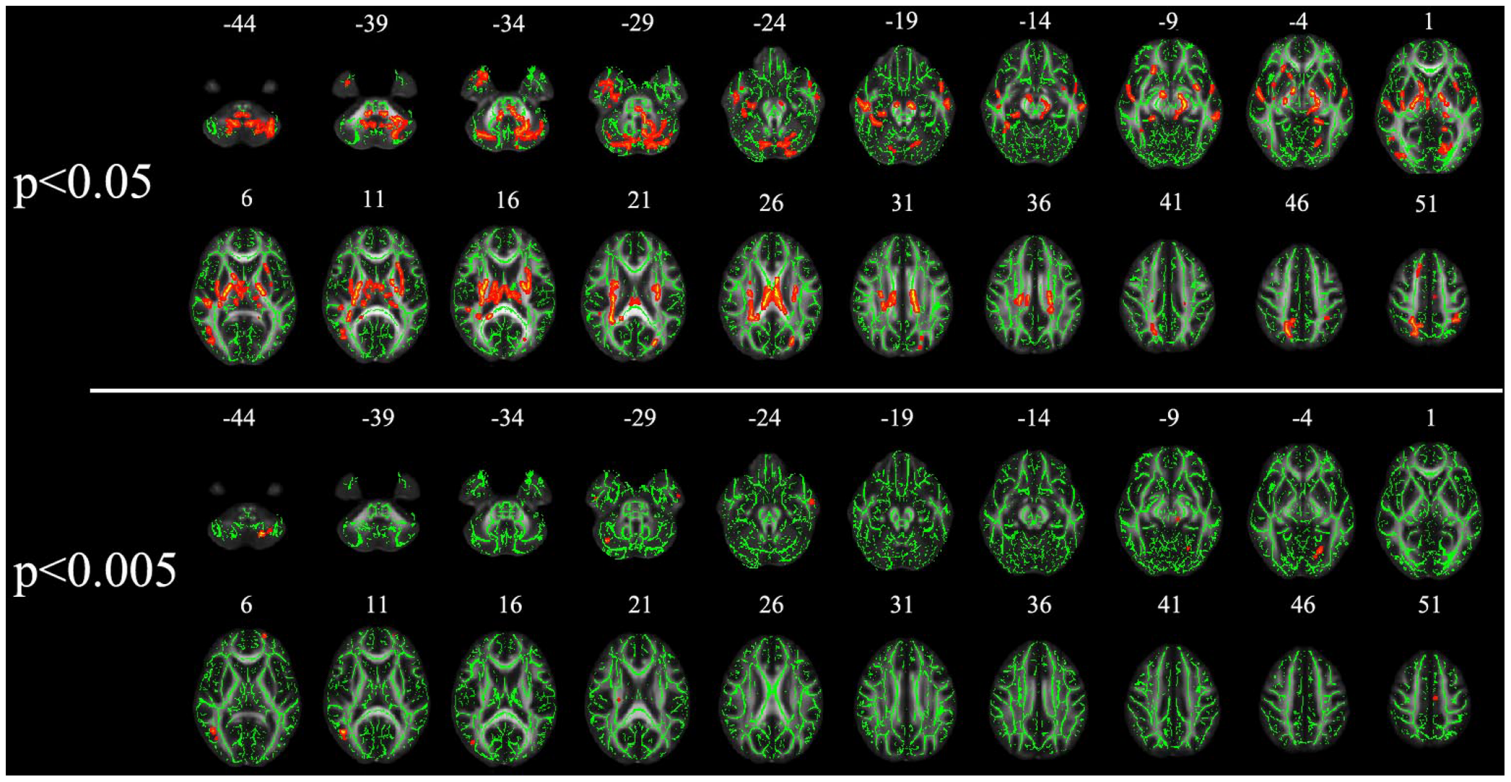
Statistical maps of group comparison of fractional anisotropy (FA) value on a voxelwise basis. The group’s mean FA skeleton (green) was overlaid on the Montreal Neurological Institute template. The threshold of mean FA skeleton was set at 0.2. Sea level immigrants show significantly higher FA value than high altitude residents (p<0.05 and p<0.005, uncorrected).

SL immigrants had significantly higher FA in a broad range of brain areas compared with controls (p<0.05 and p<0.005, uncorrected for multiple comparisons) (Figure 3, Table 3). The significant regions (p<0.05, clusters size >100 mm^3^) included the bilateral superior longitudinal fasciculus, bilateral inferior longitudinal fasciculus, bilateral inferior fronto-occipital fasciculus, posterior body of corpus callosum, bilateral superior corona radiata, bilateral posterior cingulum, bilateral anterior thalamic radiation, bilateral corticospinal tract, and cerebellum. The significant regions (p<0.005, clusters size>50 mm^3^) included the right inferior longitudinal fasciculus (corresponding to inferior temporal gyrus), left inferior longitudinal fasciculus (corresponding to inferior temporal gyrus and lingual gyrus), left cerebellum crus 8, right cerebellum crus 1, left superior corona radiate (corresponding to medial frontal gyrus), and corticospinal tract (corresponding to midbrain).

**Table 3:** Main regions showing greater FA in SL immigrants relative to HA controls.

MNI coordinate			Voxels (mm^3^)	White matter tract	Corresponding cortical area	FA value		p
x	y	z				SL immigrant	control	
21	22	−11	101	IFF-R	Uncinate fasciculus	0.428(0.055)	0.401(0.047)	0.024
−14	−43	2	106	Cingulum-L	Parahippocampal gyrus	0.364(0.081)	0.352(0.074)	0.018
7	−15	7	107	ATR-R	Thalamus, medial dorsal nucleus	0.293(0.053)	0.275(0.047)	0.012
−31	−37	51	110	SLF-L	Inferior parietal lobule	0.351(0.109)	0.283(0.104)	0.012
−46	11	−18	115	ILF-L	Superior temporal gyrus	0.351(0.109)	0.283(0.104)	0.012
9	−28	70	117	SCR-L	Frontal lobe	0.410(0.114)	0.374(0.107)	0.015
−21	−82	32	121	IFF-L	Precuneus	0.583(0.086)	0.525(0.087)	0.025
−33	6	−3	137	IFF-L	Insula, external capsule	0.471(0.147)	0.449(0.145)	0.023
−9	−26	−8	167	CST-L	Midbrain	0.422(0.101)	0.402(0.099)	0.005
−21	−62	−7	172	ILF-L	Lingual gyrus	0.294(0.117)	0.264(0.115)	0.002
−49	−1	−13	185	ILF-L	Superior temporal gyrus	0.270(0.081)	0.245(0.068)	0.025
−7	−11	55	210	SCR-L	Medial frontal gyrus	0.336(0.162)	0.317(0.152)	0.001
11	4	61	225	SCR-R	Medial frontal gyrus	0.376(0.131)	0.337(0.126)	0.015
56	−6	−21	241	ILF-R	Temporal lobe	0.406(0.127)	0.397(0.111)	0.01
39	−32	−23	277	ILF-R	Parahippocampus gyrus	0.278(0.087)	0.252(0.078)	0.01
14	14	−2	284	ATR-R	Anterior limb of internal capsule	0.607(0.097)	0.581(0.099)	0.017
−52	−10	−27	300	ILF-L	Temporal lobe	0.271(0.113)	0.262(0.097)	0.005
25	−11	−31	356	ILF-R	Temporal lobe	0.312(0.120)	0.303(0.114)	0.01
32	−4	−36	359	ILF-R	Limbic lobe, Uncus	0.280(0.103)	0.263(0.093)	0.009
2	0	6	422	ATR-R	Thalamus	0.325(0.077)	0.305(0.072)	0.014
18	−65	−44	480	Crus 8-R	Cerebellum	0.287(0.088)	0.271(0.077)	0.014
47	−3	−15	500	ILF-R	Insula	0.371(0.103)	0.347(0.096)	0.014
15	−10	13	544	ATR-R	Posterior limb of internal capsule	0.327(0.065)	0.306(0.063)	0.014
36	−64	−29	608	Crus 1-R	Cerebellum	0.266(0.071)	0.258(0.063)	0.004
19	−49	59	745	SCR-R	Precuneus	0.401(0.148)	0.364(0.146)	0.011
11	−25	31	1111	Cingulum-R	Cingulate gyrus	0.665(0.117)	0.628(0.121)	0.014
−6	−14	−15	1121	CST-L	Brain stem	0.606(0.138)	0.588(0.142)	0.011
35	1	1	1687	SLF-R	External capsule	0.590(0.129)	0.566(0.131)	0.012
−14	−62	−47	3123	Crus 8-L	Cerebellum	0.330(0.141)	0.314(0.131)	0.005
1	−20	−28		CC	Corpus callosum	0.271(0.117)	0.252(0.109)	0.005

ATR, anterior thalamic radiation; CC, corpus callosum; CST, corticospinal tract; ILF, inferior longitudinal fasciculus; IFF, inferior fronto-occipital fasciculus; SCR, superior corona radiata; SLF, superior longitudinal fasciculus.

### MD Values

**Figure 4:**
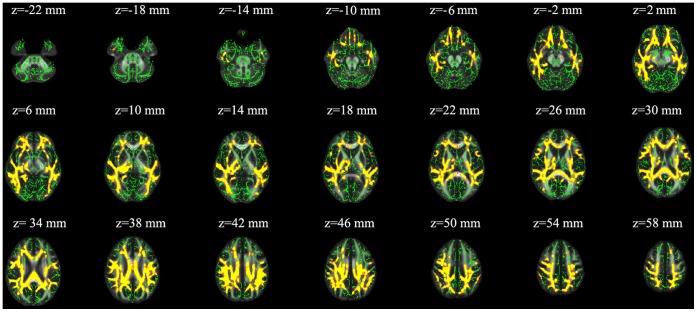
Statistical maps of group comparison of mean diffusion (MD) value on a voxelwise basis. The group’s mean FA skeleton (green) was overlaid on the Montreal Neurological Institute template. Sea level immigrants show significantly lower MD value than high altitude residents (p<0.001, corrected).

Significant decreases in MD were found in most WM tracts, except the cerebellum and the fibers in the anterior limb of internal capsule (Figure 4).

### Correlation

**Figure 5:**
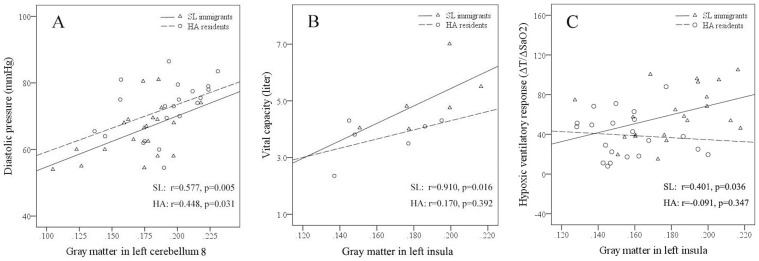
Correlations of gray matter volume with diastolic pressure and vital capacity. (A) In both sea level immigrants and high altitude residents, gray matter volume in the cerebellum lobule 8 correlated with diastolic pressure. (B) In sea level male immigrants, gray matter volume in the insula correlated with vital capacity. (C) In sea level immigrants, gray matter volume in the insula correlated with the change of tide volume (ΔT)/the change of SaO2 (ΔSaO2) ratio.

In both SL immigrants and controls, GM volume in the cerebellum lobule 8 positively correlated with diastolic pressure (Figure 5A). In SL male immigrants, GM volume in the left insula positively correlated vital capacity (Figure 5B). In SL immigrants, GM volume in the left insula positively correlated HVR test ΔT/ΔSaO2 value (Figure 5C).

## Discussion

HA adolescents immigrated to SL were at age of 11–14, which are during their brains developmental trajectory peaks [1]–[3]. Our study characterized the brain structure modulated by high level of oxygen during its peak period of development. GM volume changed in several brain regions, but little did WM volume. Significantly increased FA values and decreased MD values were observed at multiple sites of WM tracts. Moreover, GM volume in cerebellum lobule 8 positively correlated with diastolic pressure, while GM volume in insula positively correlated vital capacity and hypoxic ventilatory response. No significant difference in total GM, WM, or CSF volume was shown between adolescents grown at HA and those who lived at SL.

The regions shown changes in GM in our study have been proved to be activated by inhalation of high concentration of oxygen in previous fMRI studies. The cerebellum and insula were immediately and extensively activated by hyperoxic ventilation (100% O2) in healthy children [28]. Cerebellum and insula were also activated by 2-min hyperoxia (100% O2) in congenital central hypoventilation syndrome children (8–15 years) and compared with controls, the patients had decreased fMRI signal in the insular cortex. Moreover, fMRI signal intensity changes for the insula overlaid with breathing or heart rate traces for both patients and control groups [26]. A number of brain areas were activated during the visuospatial task. However, there was an increase of activation in the parietal lobe, frontal lobe, and cerebellum lobule under the condition of 30% than 21% oxygen [25]. Similarly, a number of brain areas were activated during the verbal task. Increased brain activations were observed in a lot of brain regions, including multiple sites of frontal cortex, with 30% oxygen administration [27]. Recently, a structural MRI study demonstrated a smaller cerebellum in mice exposed to hyperoxia (85% O2) from postnatal days 1 to 14 [18]. Histological studies on rats have shown neuronal damages in a number of regions, most prominent in the cerebellum [13], [47].

HA residents have developed adaptations in respiratory function, cardiovascular function, and brain morphology and function [9], [10], [38]–[40]. After residing at SL for a long period of time, they redevelop an adaption to high concentration of oxygen environment. For example, HA adult residents had a decrease in resting heart rate after continuous residence at SL for 2 years [31], [32]. HA residents who descended to SL over a three-month period showed a slow disappearance of electrocardiographic signs of right ventricular hypertrophy [30]. The hemoglobin in HA natives was significantly reduced during the 6 weeks at SL [42]. Increased vital capacity was found in healthy male HA residents in the third day after their arrival at SL [41]. When HA natives moved to SL, pulmonary artery pressure levels can drop to normal [29]. The pulmonary and cardiovascular changes were found in our study, showing decrease in oxygen saturation and blood pressure in both males and females and increase in vital capacity in males. At SL, through afferent feedback, the adaptation in the cardiovascular and respiratory systems may act on their control centers in the brain. This dynamic loop between brain structure and brain function is at the root of the neural basis of plasticity [48]. Therefore, we suggested, for a long term adaptation to SL environment, the changed respiratory and cardiovascular systems may act on the control centers, resulting in change of brain structure.

In addition to function-activated effects, the changed GM induced by oxygen alteration could be the result of the neurogenesis or neurodegeneration directly induced by oxygen stress. Acute exposure to high concentration of oxygen during development in animals has been long known to induce apoptosis and negatively impact neuronal cell fate [14], [16]. Hyperoxia was found changed CMRO2, which may have contribution to neurogenesis or neurodegeneration. For example, hyperbaric 100% O2 significantly increased CMRO2 [20], while 50% and 98% O2 decreased CMRO2 [21]. Aerobic glycolysis minimizes the production of reactive oxygen species in cells during the critical phases of enhanced biosynthesis and cell division [49]. However, cerebellum has significantly low levels of aerobic glycolysis compared with other brain regions [49], which may be a cause of the damage of cerebellum under hypoxic environment. In contrary, a growing body of literature has started to connect neurogenesis with hyperoxia when it is controlled at low-to-moderate levels. Hyperoxia-induced reactive oxygen species is known to modulate the redox state of tyrosine phosphorylated proteins, thereby having an impact on many transcriptional networks and signaling cascades important for neurogenesis [19], [50], [51]. Moderate level of oxygen induced neurogenesis may be involved in GM increases in the insula and posterior parietal cortex found in our study.

Vasculature accounts for about 5% of GM, and thus vascular alteration can contribute to GM changes [48]. CBF decreased ranging from 9 to 31% in response to 100% O2 administration and the decrease in CBF was greater in young adults than in older subjects [22]. Because the level of perfusion is considerably lower in WM relative to GM regions [24], this decrease in perfusion induced by hyperoxia occurred predominately in GM, with little or no measurable change in WM regions. Even within GM, the hyperoxic induced vasoconstriction depends on regions. For example, hyperoxia diminished CBF in all regions except in parietal and left hemispheric frontal GM [23]. In contrary to inducing of vasoconstriction, hyperoxia can stimulate vasculogenic stem cell growth and differentiation in vivo [52]. Reactive oxygen species is believed to be involved in vascular remodeling, such as enhancement of vascular smooth muscle growth and activation of matrix metalloproteinases, and in the alteration of vascular smooth muscle tone [53].

Generally, there is evidence for increasing FA during adolescence [1]. In 10 major WM tracts, most children had increasing FA and decreasing MD between scans, demonstrating widespread maturation [4]. Greater FA may reflect greater myelination of WM fibers, increased number of myelinated fibers, smaller axonal diameter, or reduced neural branches within MRI voxel [10]. MD quantifies the average magnitude of microscopic water diffusion, which is likely to reflect cellular density and extracellular fluid volume, and relates to the volume fraction of the interstitial space [54]. Lower MD values indicate the existence of more diffusion barriers such as cell membranes or myelin sheaths. Therefore, the higher FA accompanied by lower MD mean that the motion of water diffusion is more restricted and more directional. A number of researches indicated that oligodendroglial loss [15], [55] and myelination delay [56] caused by acute exposed to high concentration of oxygen may be related to WM damage. In another aspect, neurogenesis induced by low-to-moderate level hyperoxia has been proved in vitro and vivo observations [19]. In the present study, we found a higher FA and a lower MD at a number of WM tracts in SL immigrants, which may be related to the increase of oligodendroglial differentiation. The limitation of our study is the weak statistical power of FA value analysis because the results obtained in the TBSS analysis could not survive multiple comparison correction.

The ventilatory response to hyperoxia is called “hyperoxic hyperventilation”, and it mainly shows an increase in tidal volume, with or without change in respiratory frequency [57]. In the present study, we also found a higher vital capacity in SL immigrant. The findings from neuroimaging studies of volitional motor control of breathing converge to define a cortico-striatal-bulbar-cerebellar circuitry, which consists of the sensorimotor cortex, cerebellar hemispheres, supplementary motor cortex, and premotor cortex [58]. Posterior parietal secondary sensory cortex and insular cortex are the two primary elements of respiratory sensation [58]. The source of the respiratory-related evoked potential components P3 was located at the parietal cortex [59]. Insular cortex has been the most consistently reported structure in all the studies of respiratory sensory perception [58]. Moreover, left posterior parietal lobe and insula have also been the most consistently implicated structure in patients known to have diminished respiratory sensations [58], [60]. Our present study found that GM decreased in the regions controlling volitional motor, while increased in the regions related to respiratory sensory perception. GM volume in insula in the SL male immigrants significantly correlated with vital capacity, suggesting the increased GM in insular cortex may be directly related to hyperoxic hyperventilation. In the present study, we also found a markedly increased HVR in SL immigrants, which was in consistent with the findings in the study of Vargas et al. [44]. Moreover, GM volume in insula significantly correlated with HVR test ΔT/ΔSaO2 value, which indicated that the increased GM in insular cortex may also be related to the increase in HVR.

In the present study, GM loss in the cerebellum may be responsible for decrease in blood pressure, since cerebellum lobule 8 had a significantly positive correlation with diastolic pressure in both SL immigrants and HA residents. Cerebellum lobules 5, 6 and 8 consist of a primary sensorimotor zone, having functional connectivity with motor and premotor cortex, somatosensory, visual, and auditory cortex; Crus 2 has strong functional connectivity with the inferior parietal lobule and prefrontal cortex [61]. In our present study, GM changed in all of these cerebellar regions. We therefore presented a network consist of these regions incorporated in cardiovascular adaptation in SL environment. GM increase in the left insula may be also responsible for decrease in blood pressure. Insular cortices are involved in not only sensory representation of cardiovascular adjustments once they have been made, but also in the active modulation of efferent neural changes that elicit the cardiovascular response [62]. The left insula principally regulate parasympathetic activity [63], down-regulating blood pressure. Cerebellum and insula response to hyperoxia accompanied by increasing blood pressure [28]. A recent fMRI study showed a different BOLD response between cerebellar cortex and insula to increase of blood pressure induced by Valsalva maneuver. The left cerebellar crus 2 showed a signal increase, while the left insula exhibited a signal decrease to the Valsalva in healthy people and, in contrast, heart failure patients showed distorted signal patterns in this two regions [64].

There were several limitations in our study. The HA residents, who consisted of the control group, had moved to lowland for 1–15 days, which would have an influence on CBF. However, given the previous investigations, this affect may be slight after residence at SL for such a short period of time. For example, the removal of adult natives from HA to SL for 6 weeks resulted in only minor changes to the cardiac structure and function [42]. When HA adult natives entered SL, their cardiac index remained unchanged [31]. Except for suffering from high level of oxygen, HA adolescents living at SL will be challenged in their emotional well-being, such as depression and stress caused by being far away from home and away from their parents, and in a little bit cultural difference. All of these stresses during development interfere with the critical waves of neurogenesis, synaptic overproduction, and pruning of synapses and receptors [65]. Diet did not appear to be an important factor in this change, because food similar to that in Qinghai-Tibetan Plateau was available to the subjects and they were able to eat without any significant alterations to their dietary habits.

In conclusion, although the influence of high concentration of oxygen at normal or higher atmospheric pressure on the development of the brain has been investigated for a long time [66], this study, for the first time, revealed the modification of brain structure by oxygen during its developmental peak periods. The brain is a highly aerobic organ with very small energy stores, making neuronal activity and energy metabolism greatly influenced by oxygen delivery. Therefore, neuronal activity, blood flow, glucose consumption, and capillary density are all tightly correlated. Due to limited resolution of MRI, the cellular processes underlying such structural changes can not be revealed by in vivo neuroimaging [67]. Our finding indicate that the developmental modulations of GM by high level of oxygen are related to respiratory and circulatory regulations, while the modulation in WM exhibits an enhancement in myelin maturation. In patients, hyperoxia therapy has been shown to be a useful tool in the treatment of neurological, psychiatric, and neurotrauma deficits [12]. Characterizing the effect of oxygen modulation on the brain structure may have implications for hyperoxic therapy in nervous system diseases and activity of HA residents at lowland.
